# Hydrodynamic entanglement in the abyss: Morphological adaptations of groups of *Euplectella aspergillum*

**DOI:** 10.1093/pnasnexus/pgag165

**Published:** 2026-05-18

**Authors:** Giacomo Falcucci, Ourania Giannopoulou, Paolo Proia, Giorgio Amati, Maurizio Porfiri

**Affiliations:** Department of Enterprise Engineering “Mario Lucertini”, University of Rome “Tor Vergata”, 00133 Rome, Italy; Department of Physics, Harvard University, Cambridge, MA 02138, USA; Department of Enterprise Engineering “Mario Lucertini”, University of Rome “Tor Vergata”, 00133 Rome, Italy; Department of Enterprise Engineering “Mario Lucertini”, University of Rome “Tor Vergata”, 00133 Rome, Italy; High Performance Computing Department, CINECA Rome Section, 00185 Rome, Italy; Department of Biomedical Engineering, Tandon School of Engineering, New York University, Brooklyn, NY 11201, USA; Department of Mechanical and Aerospace Engineering, Tandon School of Engineering, New York University, Brooklyn, NY 11201, USA; Center for Urban Science and Progress, Tandon School of Engineering, New York University, Brooklyn, NY 11201, USA; Department of Civil, Urban and Environmental Engineering, Tandon School of Engineering, New York University, Brooklyn, NY 11201, USA

**Keywords:** fluid dynamics, glass sponges, high performance computing, hydrodynamic interactions, lattice Boltzmann method

## Abstract

*Euplectella aspergillum* is a deep-sea glass sponge that has attracted the interest of the scientific community for almost 150 years, for its surprising adaptations to the asperities of the abyss. The state-of-the-art on this organism focuses on specimens in isolation, but field observations question this premise. Footage from the abyss shows instances in which *E. aspergillum* live in bouquets comprising several organisms. Through high performance computing of the flow physics of *E. aspergillum*, we discover a complex hydrodynamic entanglement that favors downstream organisms at no cost to upstream ones. Such an interaction benefits the nutrition, reproduction, and resilience of the bouquet—the first instance of a hydrodynamic advantage that emerges due to purely passive interactions in a group.

Significance Statement Deep-sea glass sponges dwell the depths of the oceans, withstanding the harsh environmental challenges of the abyss. Specimens displayed in museums and videos taken by deep divers suggest that these organisms sometimes live in isolation and sometimes in bouquets: is there a hydrodynamic advantage to living in groups for these immobile organisms? We seek to answer to this question by resorting to extreme, high performance computing simulations, with advanced numerical schemes, unveiling part of the mysteries of these puzzling dwellers of the abyss.

## Introduction

Hydrodynamics is a determinant of life in the deep ocean (1). The combination of high mass density, high viscosity, circulatory motions, and carrying capacity (salt, heat, etc.) pose unique challenges for marine organisms to survive (2). For many fish species, life in schools is instrumental to addressing some of these challenges (3), with recent laboratory evidence showing that schooling fish are able to halve their energy expenditure compared to fish swimming in isolation (4).

Whether and how the hydrodynamics benefit of life in groups extend to marine noninsect and invertebrate organisms are open questions, with scant literature and mixed results (5–7). For example, Sutherland and Weihs (5) have shown that salps—barrel-shaped, planktonic tunicates—arranged in chains of multiple swimming units minimize the drag associated with instantaneous velocity changes from periodic jet propulsion. In contrast, computational results by Samson et al. (6, 7) do not support that Xeniid corals—a family of soft corals—benefit from coordinated pulsing, thereby hinting to nonhydrodynamic factors underpinning life in groups, such as predator avoidance and defense. Let that be the swimming in a chain or pulsing in a group, the search for hydrodynamic benefits has always revolved around the premise of regulating some form of relative activity among individual organisms. Can hydrodynamic benefits emerge due to purely passive interactions in a group?

We seek to answer to this question through the study of passive hydrodynamic interactions between *Euplectella aspergillum* (often referred to as “Venus flower basket”), a dweller of the deep sea whose morphological adaptations continue to amaze the scientific community (8–15). Unique to these sponges is their glass skeleton, comprising siliceous *spiculae* arranged in a lattice pattern and decorated by helical ridges that protrude out of the sponge body (16). Recent research has shown that their skeletal structure is optimized not only from a structural point of view (17), but also from a hydrodynamic perspective (12, 14), lending benefits to this organism for its nutrition, reproduction, and resilience.

Computer simulations (12) of isolated organisms immersed in a fluid flow unveiled a complex wake structure, in the form of a quiescent zone close to the sponge that develops into a region of intermittency away from the organism. Such a wake helps reduce the drag and lift experienced by the organism (12), enhancing torsional resistance and radial stiffening of the skeletal structure (18). Within the body cavity, one observes the emergence of spiraling upward motions toward the *osculum*, which enhance the sponge filtration capability (19) and favor gamete encounters for sexual reproduction (12). In a completely passive manner, the sponge is able to scavenge its resources from the surroundings over a broad range of adverse flow conditions (14).

**Fig. 1.**
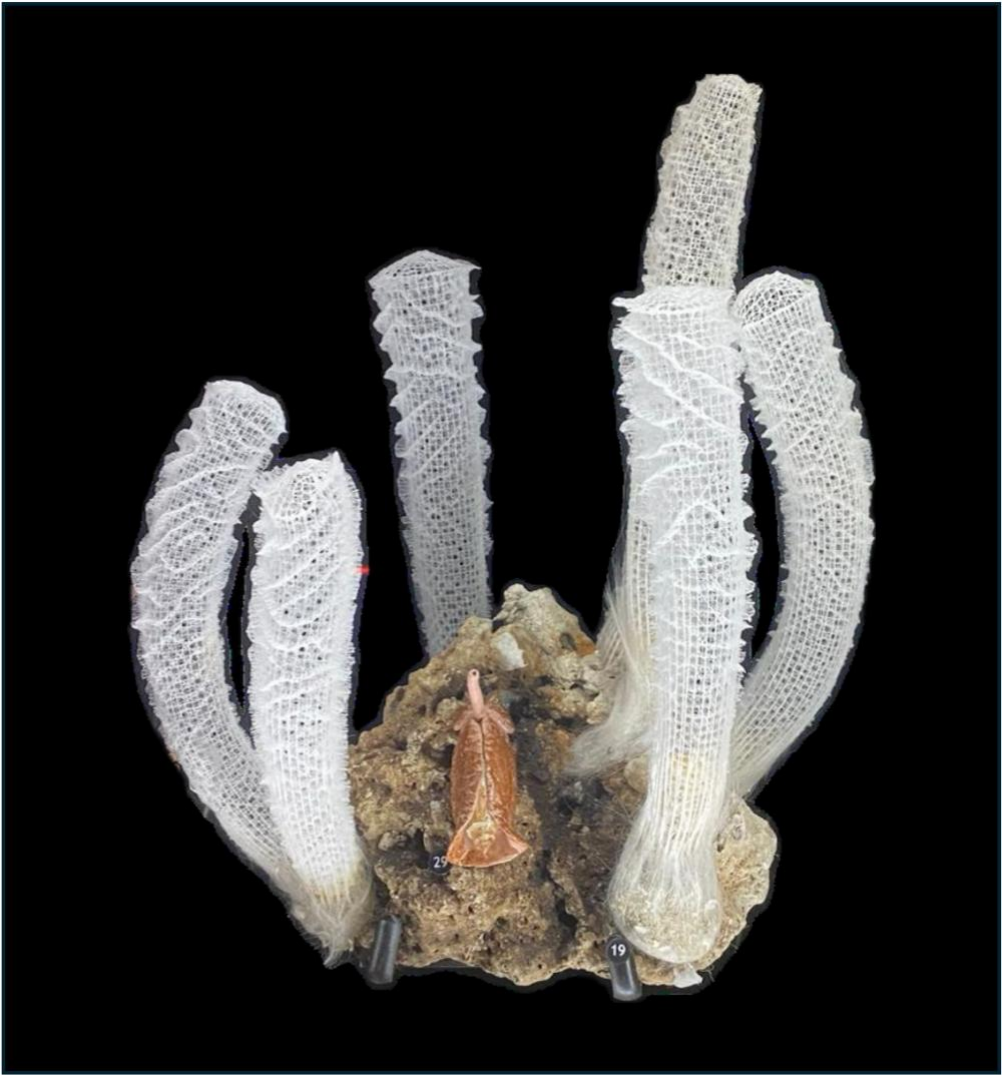
Photo of a *bouquet* of *E. aspergillum* from the George W. Strake Hall of Malacology, at the Houston Museum of Natural Sciences in Texas, USA; image taken by M. Porfiri.

Evidence of life in a *bouquet* for *E. aspergillum* dates back to the late nineteenth Century, as documented by T.J. Moore, who described the process of retrieving specimens by a local fisherman who “haul[s] his line gently in, and generally finds two or three Regaderas [*E. aspergillum*] impaled on the hooks.” Museum installations (Fig. 1), footage from the abyss (20), and recent reports from scientific explorations (21, 22) make the clues of T.J. Moore evident.

## Results

### Computational framework

**Fig. 2.**
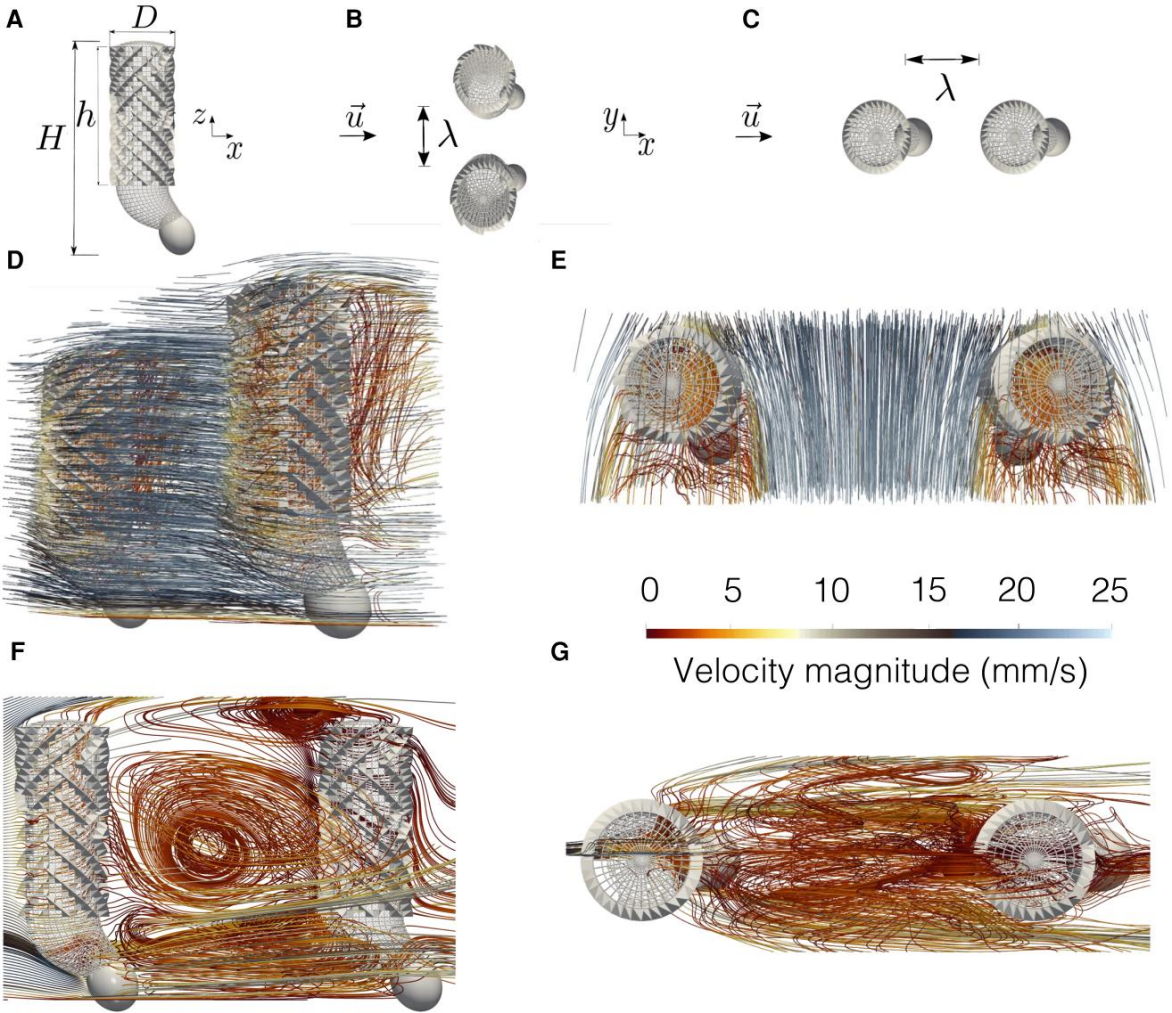
HPC simulations of hydrodynamic interactions between pairs of *E. aspergillum* sponges. A) Morphology of the digital twin of *E. aspergillum*, with H=154mm being the height of a specimen from the bulb to the *osculum*, D=40mm the inner diameter of the body cavity, and h=100mm the height of the body cavity. B) SBS configuration: the two sponges are perpendicular to the flow direction and separated by a distance *λ* (λ=D,  3D, and 5D). C) IL configuration: the two sponges are along the flow direction and separated by a distance *λ* (λ=D,  3D, and 5D). D, E) Front and top views of flow pathlines for the SBS configuration at Re=500 and λ=3D. F, G) Front and top views of flow pathlines for the IL configuration at Re=500 and λ=3D. Pathlines are colored according to the magnitude of the flow velocity, shown in the common color legend.

We explore hydrodynamic interactions in a pair of sponges through high performance computing (HPC) simulations with the lattice Boltzmann method (LBM) (23) (see Methods). Each digital sponge is H=154mm tall and D=40mm wide, with a space resolution of $2\times 10^{-4}\;m$ (Fig. 2A). Our digital twin of *E. aspergillum* resolves ridges and spicule-scale features, as explained in Ref. (12).

We consider two different layouts—side-by-side (SBS) (Fig. 2B) and in-line (IL) (Fig. 2C)—based on the orientation of the sponges with respect to the incoming flow velocity $u_{\mathrm{in}}$. We conduct a parametric analysis by varying the relative distance between the sponges, λ=D,  3D, and 5D, and incoming flow conditions, corresponding to Reynolds numbers Re=|uin|D/ν=500,  1,000, and 2,000 (for kinematic viscosity, we employ the value reported in Ref. (24): $\nu =1.75\times 10^{-6}\;m^{2}\;s^{-1}$). In addition to sponges in pairs, we also reexamined the simulations on sponges in isolation performed in Refs. (12, 14) to extract any measure needed for comparison (see Methods).

The computational requirements for such a quest are extreme; we resort to Leonardo, the leading HPC facility of the Italian Institution for super calculus CINECA (25) that ranks 9th among the top supercomputers in the world (26). We simulate domains larger than $10^{11}$ computational sites, through a massively parallel implementation of the LBM that leverages both CPU parallelization and GPU acceleration (see Methods). Simulation results indicate stark differences in the flow physics between the two configurations: while the interactions between the two sponges seem to be minimal in the SBS configuration (Fig. 2D and E for Re=500 and λ=3D; other cases are shown in Figs. S1–S3), we register a dramatic coupling for IL sponges (Fig. 2F and G for Re=500 and λ=3D; other cases are shown in Figs. S4–S6). Therein, the downstream sponge dwells in a nearly quiescent region, created by the presence of the upstream sponge. A hydrodynamic entanglement emerges between the two organisms, with the upstream sponge shielding the downstream organism and vertically redirecting the incoming streamwise flow.

Toward quantitatively assessing the role of hydrodynamic interactions on life in groups, we study both the flow physics within the body cavities and the hydrodynamic load experienced by the organisms for the two configurations.

### Flow physics within the body cavities

**Fig. 3.**
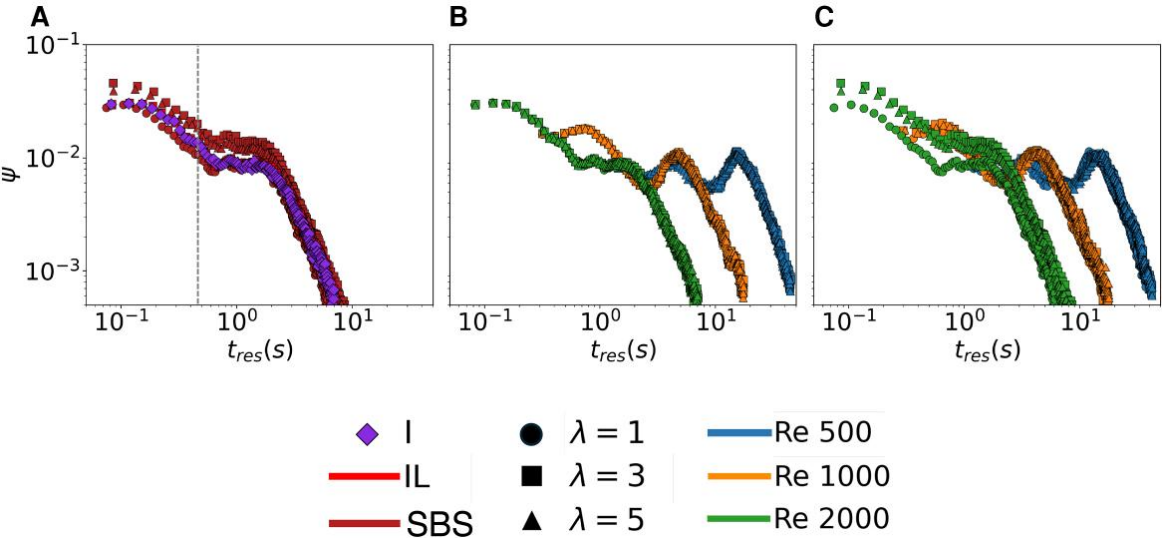
Living upstream of or on the side of another sponge does not challenge the ability of an organism to feed and reproduce. A) Comparison between the distribution of the residence times between an isolated sponge (I), an upstream sponge in the IL configuration, and the right sponge with respect to the flow direction in the SBS configuration, at Re=2,000, for λ=D,3D, and 5D. The dashed gray line represents the reference “ballistic” cross time $t_{\mathrm{cross}}=\frac{D}{\left|u_{\mathrm{in}}\right|}∼0.45$ s at the considered flow regime. B) Distribution of the residence times in the upstream sponge in the IL configuration at Re=500,1,000, and 2,000 for λ=D,3D, and 5D. C) Distribution of the residence times in the right sponge, with respect to the flow direction, in the SBS configuration at Re=500,1,000, and 2,000 for λ=D,3D, and 5D.

With respect to the flow within the body cavities, we extracted all the pathlines that are fully contained inside each body cavity and we computed their residence time, $t_{\mathrm{res}}$, defined as the time a fluid particle requires to travel along the full length of the pathline $l_{\mathrm{path}}$ (see Methods). By aggregating data for all the pathlines within each sponge, we created statistical distribution, $\tilde{\psi }$, for the residence time which is informative of the time made available to each sponge for filter feeding and sexual reproduction. The wider the distribution, the more effective a sponge is in passively scavenging nutrients from their surroundings (27) and increase the likelihood of gamete encounters in its body cavity (28). Irrespective of the distance between the sponges and the Reynolds number, the flow within the body cavity of the upstream organism in the IL configuration is equivalent to that of an isolated sponge across different Reynolds numbers (Figs. 3A, S7, and S8). As such, the distance between the sponges has no effect on the distribution of the residence time within the upstream sponge, which is fully controlled by the Reynolds number: the higher the Reynolds number, the lower the residence time (Fig. 3B).

Results for SBS sponges are analogous, whereby the distance between the sponges does not affect the statistical distribution of the residence times within each body cavity, which is controlled by the Reynolds number for both the sponges (Figs. 3C and S9). Equivalent conclusions can be drawn from the analysis of the pathline lengths inside each body cavity (Figs. S10–S13).

**Fig. 4.**
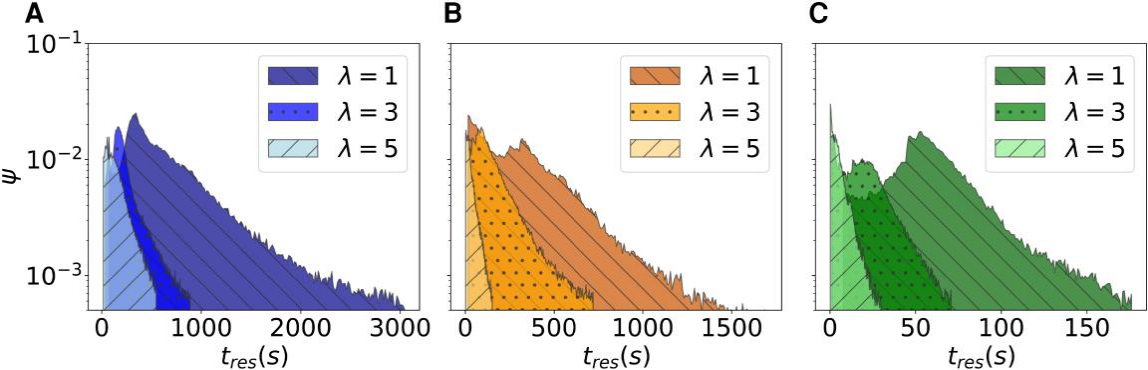
Living downstream another sponge improves the ability of an organism to feed and reproduce. A) Distribution of the residence times in the downstream sponge in the IL configuration at Re=500 for λ=D,3D, and 5D. B) Distribution of the residence times in the downstream sponge in the IL configuration at Re=1,000 for λ=D,3D, and 5D. C) Distribution of the residence times in the downstream sponge in the IL configuration at Re=2,000 for λ=D,3D, and 5D.

Whether a sponge is upstream or on the side of another sponge, there is no downside in life in a group. The upside of life in a group is in the remarkable increase in the residence times experienced by the downstream sponge in the IL configuration (Fig. 4) with respect to an isolated organism (Figs. 3A, S7, and S8) or an organism that is upstream (Fig. 3B) or on the side (Figs. 3C and S9) of another one – equivalent claims can be drawn by examining the pathline lengths (Figs. S10–S14). Decreasing the distance between the sponges in the IL configuration widens the distribution of the residence times, with up to 5-fold gains for λ=D as compared to 5D, for Re=1,000 and 2,000. For the smallest Reynolds number, Re=500, the extent of the widening is 2-fold, likely due the lower inertia of the fluid flow downstream the first sponge, which challenges the penetration of the flow through the body cavity (14). Further analysis confirms that the residence time in the downstream sponge is typically ten times larger than the time a fluid particle would take to travel a length equal to the diameter of the body cavity at a speed given by the inlet velocity (Table S1). To exclude the possibility that the increased residence time would correspond to a nearly stagnant flow that would not facilitate mixing within the body cavity, we computed the flow rate entering the body cavity of the downstream sponge (Table 1). Predictably, the flow rate increases with the Reynolds number, due to higher flow speed, and it increases with the relative distance between the sponges due to a lower shielding effect from the upstream sponge. As compared to a sponge in isolation living in an equivalent Reynolds regime, the downstream sponge benefits from hydrodynamic coupling, experiencing as much as a 10-fold increase in the flow rate. Such an investigation confirms that the fluid flow within the downstream body cavity is characterized by a strong spiraling motion that is conducive to feeding and reproduction.

**Table 1.** Living downstream another sponge enhances the flow rate in the body cavity.

Re	$\frac{\lambda }{D}$	IL	$Re_{local}$	Flow rate variation
(−)	(−)	downstream (L/h)	(−)	compared to (14) (%)
500	1	1.08	17	373.62 to 932.95
	3	2.69	43	307.09 to 1,073.3
	5	3.56	78	113.25 to 439.59
1,000	1	0.78	29	79.09 to 416.14
	3	3.29	51	96.85 to 397.95
	5	9.79	169	123.17 to 651.03
2,000	1	4.63	192	− 17.62 to 177.25
	3	22.90	153	232.33 to 1,018.4
	5	48.15	503	− 37.63 to 77.01

Reynolds number (first column), relative distance between the sponges in the ILconfiguration (second column), flow rate in the sponge downstream (third column), local Reynolds number computed based on the velocity magnitude at 0.4mm upstream of the sponge ridges (fourth column), and flow rate variations in the sponge downstream with respect to the case of an isolated sponge dwelling in a similar Reynolds regime (fifth column) . When estimating the flow rate in an isolated sponge, we utilize values reported in Ref. (14) for the two Reynolds numbers bounding the recorded local Reynolds number. For example, when estimating the flow rate for an isolated sponge in the first row of the table that experiences a local Reynolds number of 17, we utilize flow rates at Reynolds numbers of 10 and 20 that are available from Ref. (14).

To a lesser extent, hydrodynamic interplay also benefits the flow rate of SBS sponges (Table 2), whereby we document a sizable increase in the average flow rate in the pair as compared to a sponge in isolation. Such an advantage is likely associated with a convergent effect: as the sponges become closer and closer, the gap flow between the sponges facilitates water admission into the body cavities (Fig. S15). The variation of the average flow rate decreases with the Reynolds number, in agreement with prior observations on isolated sponges (14) that established a connection between the viscous length of the flow and the size of the skeletal motifs. At viscous lengths larger than the size of the pores, the sponge acts as a solid obstacle. Decreasing the viscous length favors the penetration of the flow inside the body cavity, provided that the flow is sufficiently slow for the helical ridges to divert it upwards.

**Table 2.** Living SBS another enhances the flow rate in the body cavity.

Re	$\frac{\lambda }{D}$	SBS	SBS	Average flow rate variation
(−)	(−)	left (L/h)	right (L/h)	compared to (14) (%)
500	1	33.24	30.71	17.55
	3	29.96	29.61	9.55
	5	29.11	27.87	4.74
1,000	1	91.28	86.95	15.43
	3	82.45	82.32	6.72
	5	78.93	79.65	2.70
2,000	1	226.37	220.60	13.44
	3	203.83	210.68	5.21
	5	198.41	203.34	1.60

Reynolds number (first column), relative distance between the sponges in the SBS configuration (second column), flow rate in the sponge on the left with respect to the incoming flow (third column), flow rate in the sponge on the right with respect to the incoming flow (fourth column), and average flow rate variation in the sponges with respect to the case of an isolated sponge (fifth column). When estimating the flow rate in an isolated sponge, we utilize values reported in Ref. (14).

### Hydrodynamic loading

**Fig. 5.**
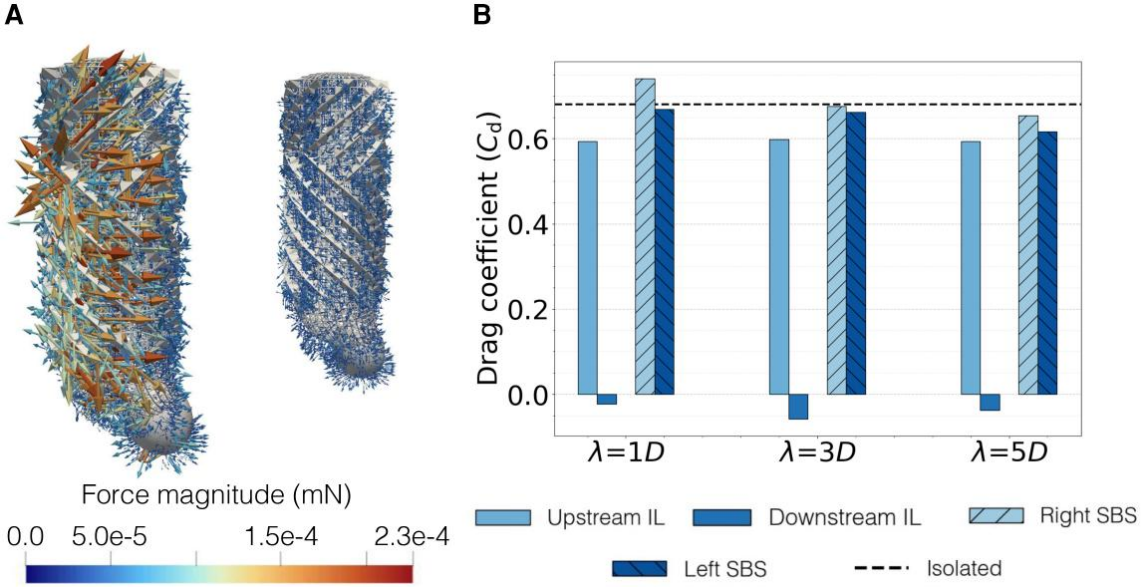
Living downstream another sponge abates the drag that an organism experiences. A) Forces acting on the downstream and upstream sponges in the IL configuration at Re=2,000 and λ=D. Each vector is the force at a lattice grid point on the solid surface. B) Bar graph of the drag coefficient versus *λ* for the IL and SBS configurations at Re=500. The dashed line refers to the drag coefficient of an isolated sponge.

To quantify the hydrodynamic loading experienced by the sponges, we computed the traction at every computational site on the sponge surface (Fig. 5A; see Methods and Figs. S16A and S16B). From the distribution of the traction fields, we extracted the drag on each sponge for both configurations and all the values of Re and *λ* as time series and computed time averages—time variations were modest for all cases, supporting that simulations reached the statistical steady state (Fig. S17).

Whether a sponge is upstream or on the side of another sponge, there are no substantial differences in the drag it experiences with respect to an organism in isolation (Fig. 5B and Tables S2–S4). Specifically, the drag experienced by the upstream sponge in the IL configuration is indistinguishable from the drag on an isolated sponge. This finding rests upon the flow regime in which the sponges dwell, in which compressibility effects are negligible and inertia plays a key role (1). The drag experienced by any of the two sponges in the SBS configuration is 10–20 higher than the drag on an isolated sponge at the same Reynolds number. When the sponges are close to each other, they virtually behave as a single porous body with a cross-sectional area larger than the areas of the two sponges, so that the drag on each sponge is magnified by the proximity to the other. The extent of the coupling between the sponges depends on the Reynolds number, which controls the viscous length-scale of the flow; the higher Re, the less the coupling between the sponges due to the gap flow. Similar observations have been gathered in classical studies on SBS solid cylinders through simulations and experiments (29–31).

Living downstream of another sponge results in a 10- to 100-fold reduction in the drag (Fig. 5B and Tables S2, S3, and S4). Such a remarkable hydrodynamic benefit is due to the hydrodynamic entanglement between the two sponges, which abates the flow velocity in the vicinity of the sponge downstream, akin to classical observations on solid cylinders IL (29, 32, 33). Surprisingly, the drag reduction could lead to a thrust force (negative drag), where the downstream cylinder is pushed toward the upstream one.

The observation of thrust force is in agreement with the literature on cylindrical bodies aligned in a flow (29, 34), which explains such a paradoxical loading condition as a result of wake interference produced by the two cylinders. For cylinders that are sufficiently close to each other, the free shear layers from the upstream cylinder do not reattach on the downstream cylinder. As a result, the flow physics is controlled by the upstream cylinder and the downstream one is in a positive pressure gradient that creates the observed thrust force. The same mechanism is likely to underpin the creation of a thrust on the downstream sponge, albeit mitigated by the complex morphology of the skeletal structure that delays the wake of the upstream body (12). Such a delay explains the observation of a thrust on the downstream sponge even at λ=5D, in contrast with solid cylinders.

## Conclusions

In the deep ocean, sponges are subjected to water currents of varying strength and orientation (35, 36). Considering a pair of sponge as the simplest aggregate to explore the potential hydrodynamic underpinnings of life in a group, a sponge will be sometimes upstream, downstream, or on the side of another one.

Our computational results indicate that being upstream of another sponge bears no disadvantage in nutrition, reproduction, and resilience with respect to being isolated. Being downstream of another organism shields the sponge from water current, dramatically reducing the drag by one to two orders of magnitude. Such a drag reduction does not preclude feeding and nutrition, whereby the upstream sponge originates a highly perturbed, low-speed fluid region that promotes water admission to the body cavity of the downstream organism. Being on the side of another sponge produces minimal increases in the hydrodynamic loading, but it enhances nutrition and reproduction through higher water admission. Thus, living in a group bestows to *E. aspergillum* multiple hydrodynamic advantages, emerging from purely passive interactions between the organisms. While the resolution of our computational framework allows for studying hydrodynamic features that were nearly impossible to detail only a few years back, we warn caution in the over-interpretation of some of our claims. Our simulations do not consider the presence of any soft tissues, thereby failing to capture any active pathway for the sponge to regulate the flow in its surroundings, whether within its body cavity or in the wake (37, 38). A living sponge has its skeleton inside the tissue—an aspect that we cannot simulate at this stage. Future work may consider the inclusion of ad hoc boundary conditions that may capture the role of soft tissues, drawing inspiration from phenomenological insight in Ref. (39).

Some lessons may be learned from this deep-sea dweller with respect to the design of above ground and underwater engineering structures that may profit from fluid-mediated interactions with their surroundings. For example, we envision the design of sustainable high-rise buildings inspired by the morphology of *E. aspergillum* that can endure high wind loadings and promote internal air circulation.

## Methods

### HPC simulations

Numerical simulations were performed on the CINECA supercomputing facility Leonardo (25). The core parameters for the simulations are listed in Tables S5–S7. The computational domain is adjusted according to the physical layout of the sponges (isolated, IL, or SBS). Each domain is characterized by dimensions $L_{x}\times \;L_{y}\times \;L_{z}$. Simulations on isolated and IL organisms are conducted in a domain of 8,000×4,400×2,400 grid points ($85\times 10^{9}$ grid points in total), exploiting 660 GPUs. The SBS simulations are conducted on larger domains of 8,000×5,600×2,400 grid points ($107.5\times \;10^{9}$ grid points in total), exploiting up to 880 GPUs. The simulations require up to $∼5\times 10^{6}$ time steps, producing ∼150 terabytes of raw, binary data.

### Lattice Boltzmann method

The LBM is a computational fluid dynamics technique rooted in kinetic theory (40–43). The LBM algorithm is both explicit and local, facilitating parallel implementation and efficient handling of complex boundary conditions (44). Specifically, the LBM simulates the macroscopic fluid behavior through the evolution of a particle distribution function f(x,ξ,t) representing the probability density function of finding a fluid particle at position x and time *t* with velocity ξ. We discretize the temporal evolution of the distribution function by considering predetermined velocity directions along a stencil, that is,


$$f_{i}\left(x+c_{i}\Delta t,t+\Delta t\right)-f_{i}\left(x,t\right)=\Omega_{i}\left(\;f_{i}\left(x,t\right),f_{i}^{\mathrm{eq}}\left(x,t\right)\right)\;,$$

where $c_{i}$ is the *i*th discrete velocity vector along the stencil (in our work D3Q19), and Δt is the discrete time step. Here, $\Omega_{i}$ is the collision operator, which is equal to $-\frac{\Delta t}{\tau_{f}}(f_{i}(x,t)-f_{i}^{\mathrm{eq}}(x,t))$ in the BGK formulation (23). The relaxation time $\tau_{f}$ is related to the kinematic viscosity *ν* through the so called Chapman–Enskog expansion, typically as $\nu =c_{s}^{2}(\tau_{f}-\frac{\Delta t}{2})$, with $c_{s}$ being the lattice speed of sound. The local equilibrium distribution function $f_{i}^{\mathrm{eq}}(x,t)$ depends on the macroscopic density, $\rho (x,t)=\sum_{i}f_{i}(x,t)$, and velocity, $u(x,t)=\frac{1}{\rho (x,t)}\sum_{i}f_{i}(x,t)c_{i}$. With respect to the boundary conditions, we set inflow at a fixed velocity, based on Re, at the inlet; we implement an outflow with zero-gradient for density and velocity at the outlet; we set periodic boundary conditions on the side boundaries; we set no-slip boundary conditions in the form of bounce-back on the bottom surface and at the solid–fluid interface; finally, we set free-slip at the top of the computational domain. The scalability of LBM on GPUs is key to successfully simulate groups of *E. aspergillum*.

### Digital twin of *E. aspergillum*

We realize a digital twin of the skeletal system of *E. aspergillum* at a spatial resolution of 0.2mm via SolidWorks and MeshLab (12). The skeletal system of *E. aspergillum* features four regions: the main body cavity, an anchoring bulb, a curved section connecting the bulb with the main cavity, and an apical sieve plate, called *osculum*. Excluding the bulb, the skeletal system comprises: (i) a periodic checkerboard lattice of orthogonal struts of 0.5mm in thickness, reinforced by thinner double-diagonal struts of 0.2mm in thickness and (ii) a quasiperiodic arrangement of helical ridges of 5mm in thickness.

### Flow physics inside the body cavity

The residence time within the body cavity is the average time a fluid particle takes to enter and leave the cavity of the organism. As a first step, we track the pathlines inside the body cavity using ParaView. Pathline data are processed with Python in order to evaluate the residence time and the length of each pathline. For each pathline, we estimate the length and residence time by discretizing the pathline in *n* steps and summing over a discrete abscissa *s*, as follows:


$$l=\sum_{s}^{n}\|x(s)-x(s-1)\|$$

$$t_{\mathrm{res}}=\sum_{s}^{n}\frac{\left\|x(s)-x(s-1)\right\|}{\frac{1}{2}\|u(s)+u(s-1)\|},$$

where x(s) and u(s) are the location and velocity of the pathline at *s*, respectively.

### Volumetric flow rate

We evaluate the rate of the flow entering *E. aspergillum* by identifying the flux through the surface of the cylinder inscribed by the body cavity. We partition such a cylinder into six surfaces: the two bases (at the bottom of the body cavity and under the *osculum*) and four equal sections of the lateral surface (Fig. S18). Through Paraview, we evaluate the total flux through each surface.

### Computation of forces

Another advantage of LBM over traditional methods is the direct evaluation of hydrodynamic forces from the distribution function. Specifically, the force exerted by the fluid on a solid surface is equal and opposite to the rate of change of the momentum ΔP of the fluid particles interacting with the wall, which is given by


$$\Delta P=\sum_{x_{i}^{w}}\left(\;f_{i}^{\mathrm{in}}+f_{\bar{i}}^{\mathrm{out}}\right)c_{i}\;\Delta x^{3}\;,$$

where $x_{i}^{w}$ represents the solid node location; $f_{i}^{\mathrm{in}}$ and $f_{\bar{i}}^{\mathrm{out}}$ are the incoming and bounced back populations, respectively; $c_{\bar{i}}=-c_{i}$ identify the links between the fluid and solid nodes; and Δx is the lattice spacing. Note that the presence of $\Delta x^{3}$ ensures we obtain a momentum variation instead of a momentum density (40). The total force F acting on the solid is given by $F=\frac{\Delta P}{\Delta t}$, with Δt=1 being the unit time interval in the LBM algorithm. From the overall force, we compute the drag, $F_{d}$, and the lift, $F_{l}$; the former being the component acting along the incoming flow, and the latter the component perpendicular to the incoming flow and parallel to the seafloor. From these components, we calculate the drag and lift coefficients,


$$C_{d}=\frac{F_{d}}{\frac{1}{2}\rho |u{|}^{2}A},$$

$$C_{l}=\frac{F_{l}}{\frac{1}{2}\rho |u{|}^{2}A},$$

where *A* is the area of the transverse section of the sponge, perpendicular to the flow.

### Conversion of lattice to physical units

To convert lattice to physical units, we define the scaling factors for length and viscosity, δx and δν, respectively.

From the conversion parameter of the viscosity, we retrieve the scaling factor of time, δt, ensuring dynamic similitude, according to Buckingham theorem. For the forces, we also need a scaling factor of mass, which we obtain by considering the fixed density value at the outlet of the domain ($\rho_{\mathrm{out}}=1$, in lattice units).

## Supplementary Material

pgag165_Supplementary_Data

## Data Availability

All data and geometries are available at the github link https://github.com/giacomofalcucci/Euplectella_HPC and upon request to the corresponding authors.

## References

[1] Denny MW . 1990. Terrestrial versus aquatic biology: the medium and its message. Am Zool. 30(1):111–121.

[2] Herring P . The biology of the deep ocean. Oxford University Press, 2002.

[3] Weihs D . 1973. Hydromechanics of fish schooling. Nature. 241:290–291.

[4] Zhang Y, Lauder GV. 2024. Energy conservation by collective movement in schooling fish. Elife. 12:RP90352. 38375853 10.7554/eLife.90352 PMC10942612

[5] Sutherland KR, Weihs D. 2017. Hydrodynamic advantages of swimming by salp chains. J R Soc Interface. 14:20170298. 28768881 10.1098/rsif.2017.0298 PMC5582125

[6] Samson JE, Ray DD, Porfiri M, Miller LA, Garnier S. 2020. Collective pulsing in xeniid corals: Part I - Using computer vision and information theory to search for coordination. Bull Math Biol. 82:1–12. 10.1007/s11538-020-00759-2 32638174

[7] Samson JE, Miller LA. 2020. Collective pulsing in xeniid corals: Part II - Using computational fluid dynamics to determine if there are benefits to coordinated pulsing. Bull Math Biol. 82:67–88. 32474651 10.1007/s11538-020-00741-y

[8] Aizenberg J, et al 2005. Skeleton of *Euplectella* sp.: structural hierarchy from the nanoscale to the macroscale. Science. 309:275–278. 16002612 10.1126/science.1112255

[9] Brayard A, et al 2017. Unexpected early triassic marine ecosystem and the rise of the modern evolutionary fauna. Sci Adv. 3:e1602159. 28246643 10.1126/sciadv.1602159 PMC5310825

[10] Robson Brown K, Bacheva D, Trask RS. 2019. The structural efficiency of the sea sponge *Euplectella aspergillum* skeleton: bio-inspiration for 3d printed architectures. J R Soc Interface. 16:20180965. 31064257 10.1098/rsif.2018.0965 PMC6544886

[11] Fernandes MC, Aizenberg J, Weaver JC, Bertoldi K. 2021. Mechanically robust lattices inspired by deep-sea glass sponges. Nat Mater. 20:237–241. 32958878 10.1038/s41563-020-0798-1

[12] Falcucci G, et al 2021. Extreme flow simulations reveal skeletal adaptations of deep-sea sponges. Nature. 595:537–541. 34290424 10.1038/s41586-021-03658-1

[13] Blasiak R, et al 2022. A forgotten element of the blue economy: marine biomimetics and inspiration from the deep sea. PNAS Nexus. 1:pgac196. 36714844 10.1093/pnasnexus/pgac196 PMC9802412

[14] Falcucci G, et al 2024. Adapting to the abyss: passive ventilation in the deep-sea glass sponge *Euplectella aspergillum*. Phys Rev Lett. 132:208402. 38829072 10.1103/PhysRevLett.132.208402

[15] Grigoropoulos C, et al 2025. Metamaterials from the deep: optimized mechano-fluidic materials inspired by deep-sea sponges. *Preprint (Version 1) available at Research Square*.

[16] Weaver JC, et al 2007. Hierarchical assembly of the siliceous skeletal lattice of the hexactinellid sponge *Euplectella aspergillum*. J Struct Biol. 158:93–106. 17175169 10.1016/j.jsb.2006.10.027

[17] Morankar SK, Mistry Y, Bhate D, Penick CA, Chawla N. 2023. In situ investigations of failure mechanisms of silica fibers from the venus flower basket (*Euplectella aspergillum*). Acta Biomater. 162:304–311. 36963595 10.1016/j.actbio.2023.03.024

[18] Chen H, Jia Z, Li L. 2022. Lightweight lattice-based skeleton of the sponge *Euplectella aspergillum*: on the multifunctional design. J Mech Behav Biomed Mater. 135:105448. 36166939 10.1016/j.jmbbm.2022.105448

[19] Yu Y, et al 2025. A filter inspired by deep-sea glass sponges for oil cleanup under turbulent flow. Nat Commun. 16:209. 39747061 10.1038/s41467-024-55587-y PMC11696985

[20] Blue Planet II, the love story of Venus’ flower basket . https://www.facebook.com/BBCAmerica/videos/shrimp-in-a-seaweed-flower-basket/10155992501632978/.

[21] Marchió A, Jamieson AJ, Stewart HA. 2025. Through the glass ceiling: extending the depth range of glass sponges (Porifera: Hexactinellida). Mar Biol. 172:88.

[22] Ehrlich H . Extreme biomimetics. Springer, 2017.

[23] Succi S . The lattice Boltzmann equation: for complex states of flowing matter. Oxford University Press, 2018.

[24] ITTC . 2011. Fresh water and seawater properties. Recommended Procedures 7.5-02-01-03, International Towing Tank Conference.

[25] Turisini M, Amati G, Cestari M. 2024. LEONARDO: a pan-European pre-exascale supercomputer for HPC and AI applications. J Large-Scale Res Facilities. 9:1–16.

[26] https://www.top500.org/lists/top500/.

[27] Yahel G, Eerkes-Medrano DI, Leys SP. 2006. Size independent selective filtration of ultraplankton by hexactinellid glass sponges. Aquat Microb Ecol. 45:181–194.

[28] Leys SP, Mackie GO, Reiswig HM. 2007. The biology of glass sponges. Adv Mar Biol. 52:1–145. 17298890 10.1016/S0065-2881(06)52001-2

[29] Zdravkovich MM . 1987. The effects of interference between circular cylinders in cross flow. J Fluids Struct. 1:239–261.

[30] Meneghini JR, Saltara F, Siqueira CDLR, Ferrari JA Jr. 2001. Numerical simulation of flow interference between two circular cylinders in tandem and side-by-side arrangements. J Fluids Struct. 15:327–350.

[31] Surmas R, dos Santos LOE, Philippi PC. August 2004. Lattice Boltzmann simulation of the flow interference in bluff body wakes. Future Gener Comput Syst. 20:951–958.

[32] Dehkordi BG, Moghaddam HS, Jafari HH. 2011. Numerical simulation of flow over two circular cylinders in tandem arrangement. J Hydrodyn. 23:114–126.

[33] Tu J, et al 2015. Flow-induced vibrations of two circular cylinders in tandem with shear flow at low reynolds number. J Fluids Struct. 59:224–251.

[34] Ishigai S, Nishikawa E, Nishimura K, Cho K. 1972. Experimental study on structure of gas flow in tube banks with tube axes normal to flow: Part 1, Karman vortex flow from two tubes at various spacings. Bull JSME. 15:949–956.

[35] Kanari S-i, Kobayashi C, Ishikawa T. March 1991. An estimate of the velocity and stress in the deep ocean bottom boundary layer. J Fac Sci Hokkaido Univ Ser 7 Geophys. 9:1–16.

[36] Lavergne Cd, Madec G, Roquet F, Holmes RM, Mcdougall TJ. 2017. Abyssal ocean overturning shaped by seafloor distribution. Nature. 551:181–186. 29120416 10.1038/nature24472

[37] Leys SP, et al 2022. Models of flow through sponges must consider the sponge tissue. Nature. 603:E23–E25. 35322246 10.1038/s41586-021-04380-8

[38] Falcucci G, et al 2022. Reply to: Models of flow through sponges must consider the sponge tissue. Nature. 603:E26–E28. 35322244 10.1038/s41586-021-04381-7

[39] Larsen PS, Riisgård HU. 2026. Effect of ambient current on filtration rate of sponges. J Mar Sci Eng. 14:182.

[40] Krüger T, et al The lattice Boltzmann method. Vol. 10. Springer, 2017.

[41] Tiribocchi A, et al 2021. The vortex-driven dynamics of droplets within droplets. Nat Commun. 12:82. 33398018 10.1038/s41467-020-20364-0 PMC7782531

[42] Shklyaev OE, Laskar A, Balazs AC. 2023. Engineering confined fluids to autonomously assemble hierarchical 3d structures. PNAS Nexus. 2:pgad232. 37497047 10.1093/pnasnexus/pgad232 PMC10367439

[43] Fazeli A, Thakore V, Ala-Nissila T, Karttunen M. 2024. Non-Stokesian dynamics of magnetic helical nanoswimmers under confinement. PNAS Nexus. 3:pgae182. 38765716 10.1093/pnasnexus/pgae182 PMC11102084

[44] Succi S . The lattice Boltzmann equation for fluid dynamics and beyond. Clarendon, 2001.

