# Supplementary material for: Hydrodynamic entanglement in the abyss: Morphological adaptations of groups of Euplectella aspergillum
Source: PNAS Nexus. 2026 May 18;5(5):pgag165. doi: 10.1093/pnasnexus/pgag165 (PMC13220335; doi:10.1093/pnasnexus/pgag165)
Supplement: pgag165_Supplementary_Data [file pgag165_supplementary_data.pdf]

Supporting Information for

Hydrodynamic entanglement in the abyss: Morphological adaptations of groups of *Euplectella aspergillum*

Giacomo Falcucci[a,b,†,*], Ourania Giannopoulou[a], Paolo Proia[a], Giorgio Amati[c], and Maurizio Porfiri[d,e,f,g,†,*]

[a] Department of Enterprise Engineering "Mario Lucertini", University of Rome "Tor Vergata", Rome, Italy;
[b] Department of Physics, Harvard University, Cambridge, MA, USA;
[c] High Performance Computing Department, CINECA Rome Section, Rome, Italy;
[d] Department of Biomedical Engineering, Tandon School of Engineering, New York University, Brooklyn, NY, USA;
[e] Department of Mechanical and Aerospace Engineering, Tandon School of Engineering, New York University, Brooklyn, NY, USA;
[f] Center for Urban Science and Progress, Tandon School of Engineering, New York University, Brooklyn, NY, USA;
[g] Department of Civil, Urban and Environmental Engineering, Tandon School of Engineering, New York University, Brooklyn, NY, USA;
∗ Corresponding authors: giacomo.falcucci@uniroma2.it; mporfiri@nyu.edu;
† G.F. and M.P. equally contributed to this work.

This PDF file includes:

Figures S1 to S17

Tables S1 to S7

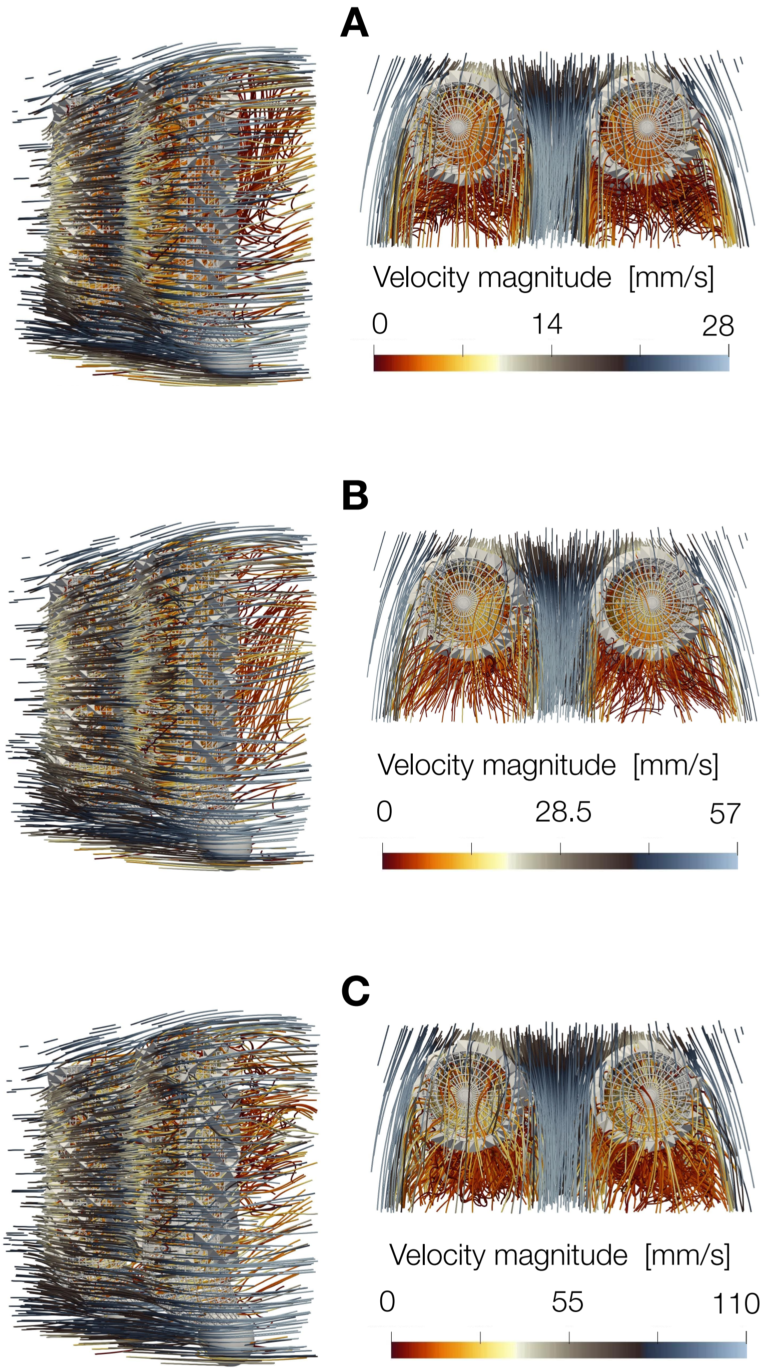

Figure S1 Flow pathlines for the SBS configuration at $\lambda = D$. Side and top views: A) $\mathrm{Re} = 500$, B) $\mathrm{Re} = 1,000$, and C) $\mathrm{Re} = 2,000$.

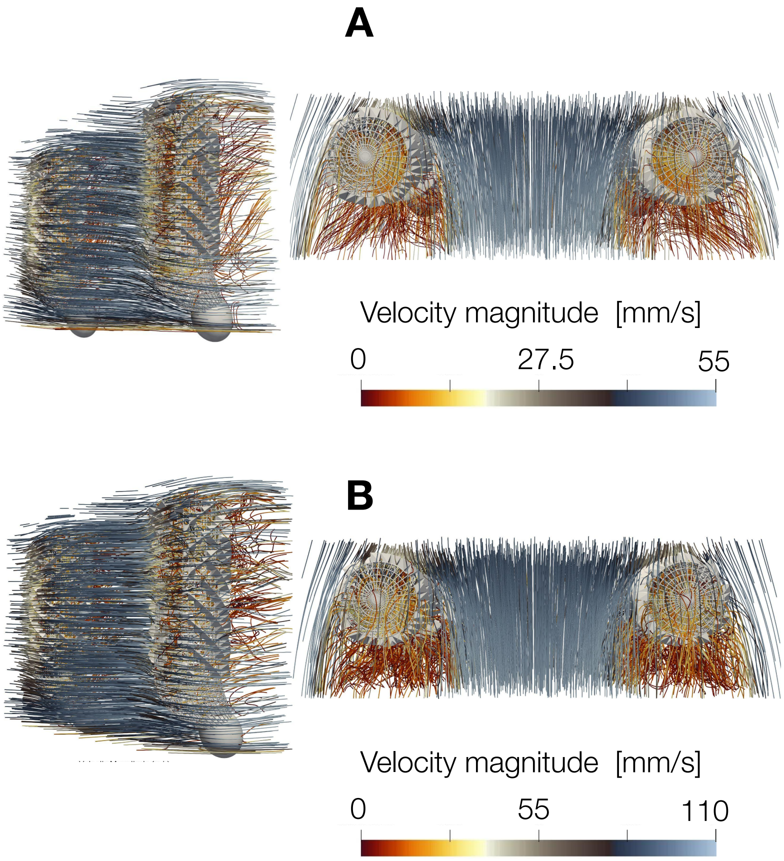

Figure S2 Flow pathlines for the SBS configuration at $\lambda = 3D$. Side and top views: A) $\mathrm{Re} = 1,000$ and B) $\mathrm{Re} = 2,000$.

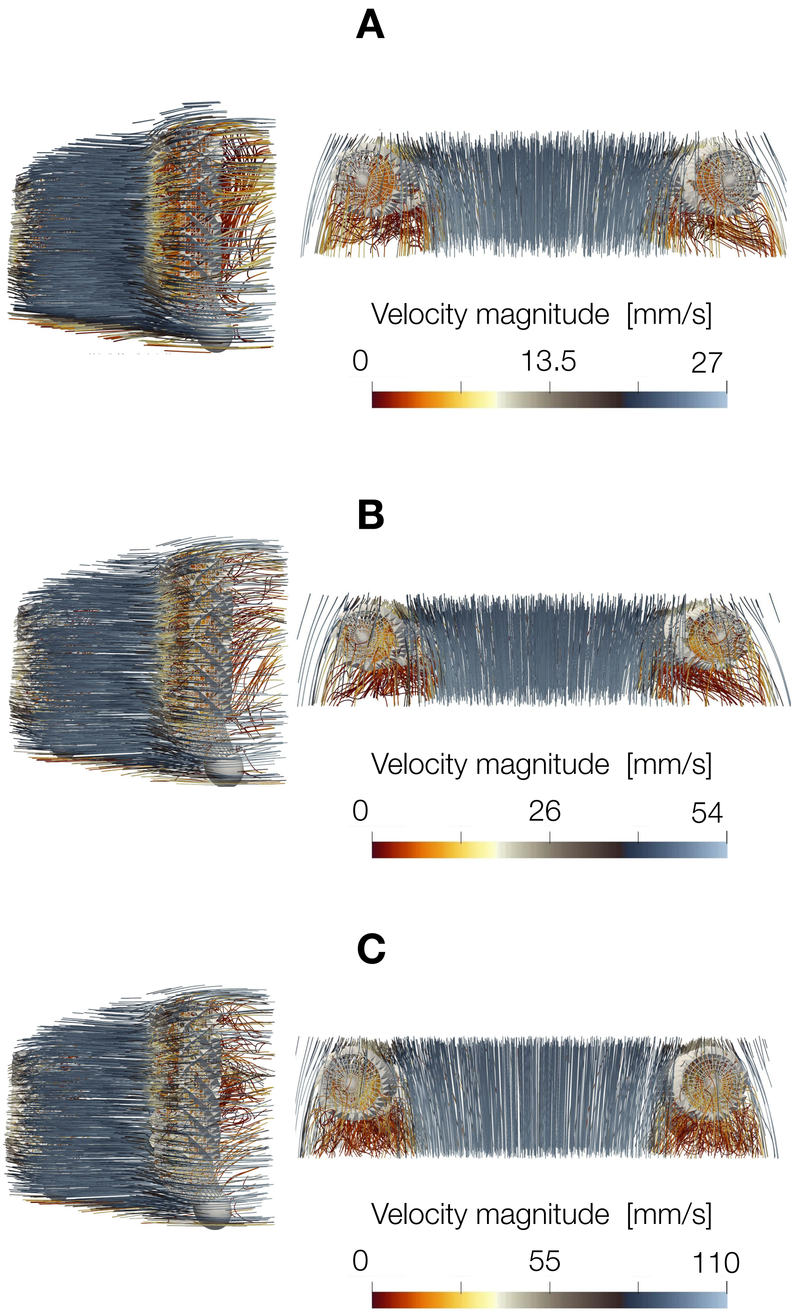

Figure S3 Flow pathlines for the SBS configuration at $\lambda = 5D$. Side and top views: A) $\mathrm{Re} = 500$, B) $\mathrm{Re} = 1,000$, and C) $\mathrm{Re} = 2,000$.

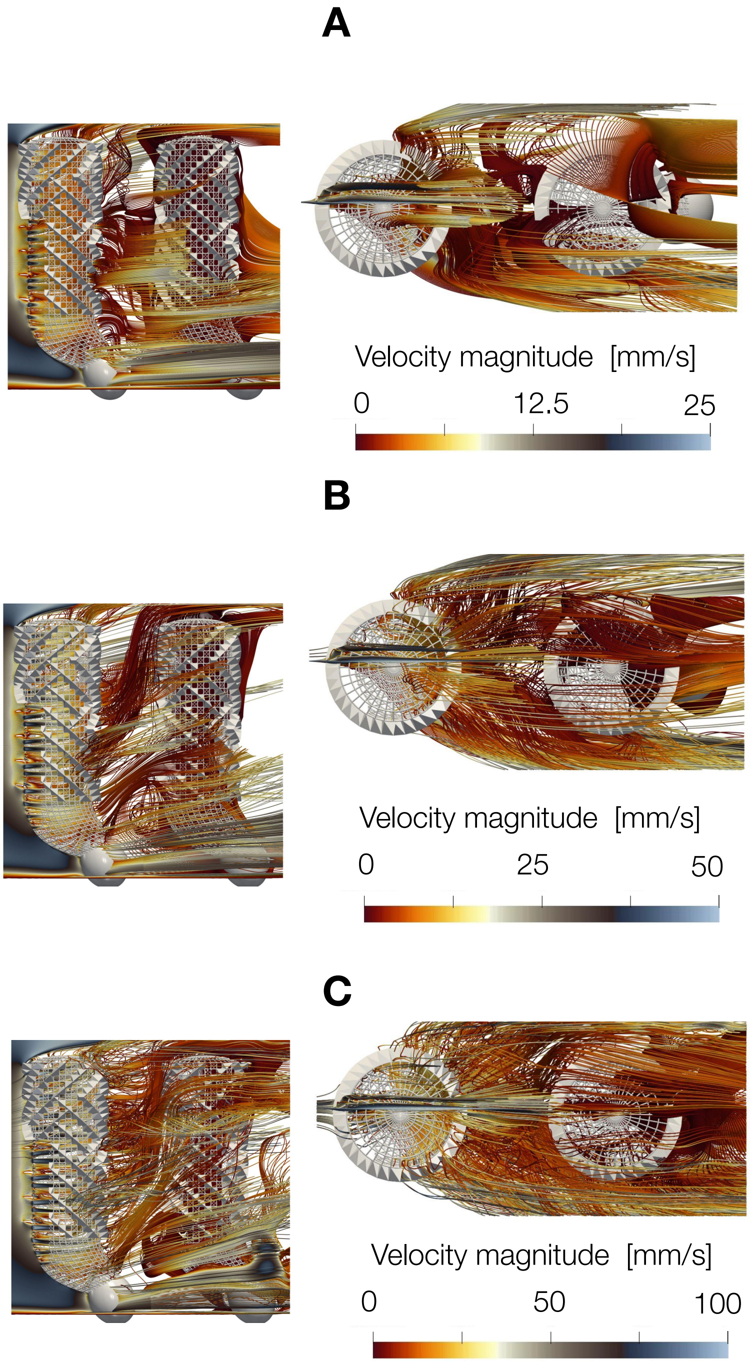

Figure S4 Flow pathlines for the IL configuration at $\lambda = D$. Side and top views: A) $\mathrm{Re} = 500$, B) $\mathrm{Re} = 1,000$, and C) $\mathrm{Re} = 2,000$.

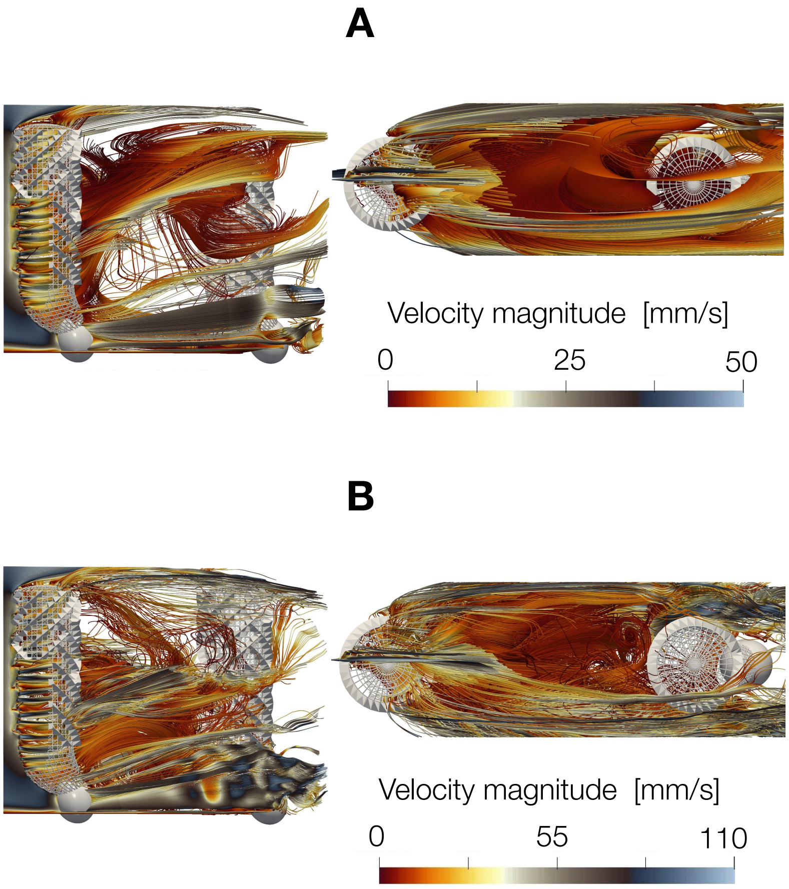

Figure S5 Flow pathlines for the IL configuration at $\lambda = 3D$. Side and top views: A) $\mathrm{Re} = 1,000$ and B) $\mathrm{Re} = 2,000$.

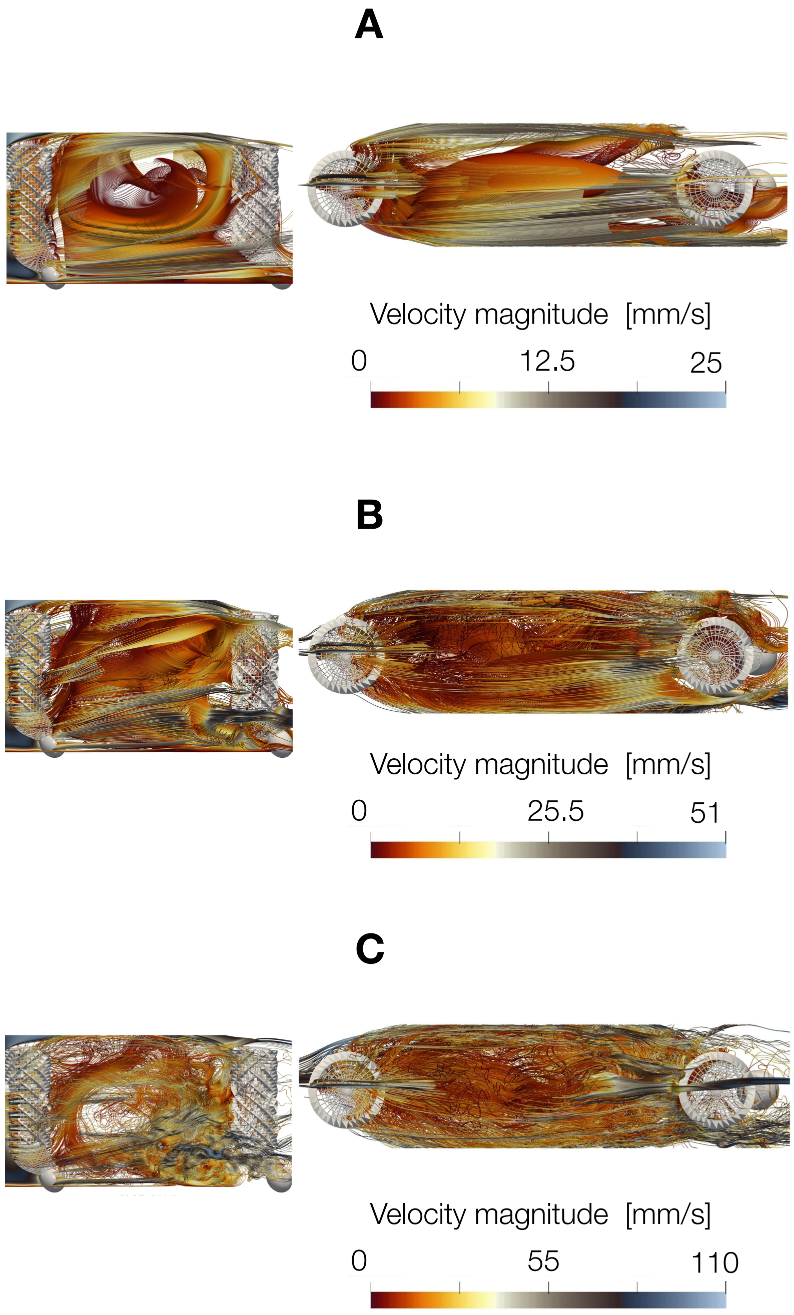

Figure S6 Flow pathlines for the IL configuration at $\lambda = 5D$. Side and top views: A) $\mathrm{Re} = 500$, B) $\mathrm{Re} = 1,000$, and C) $\mathrm{Re} = 2,000$.

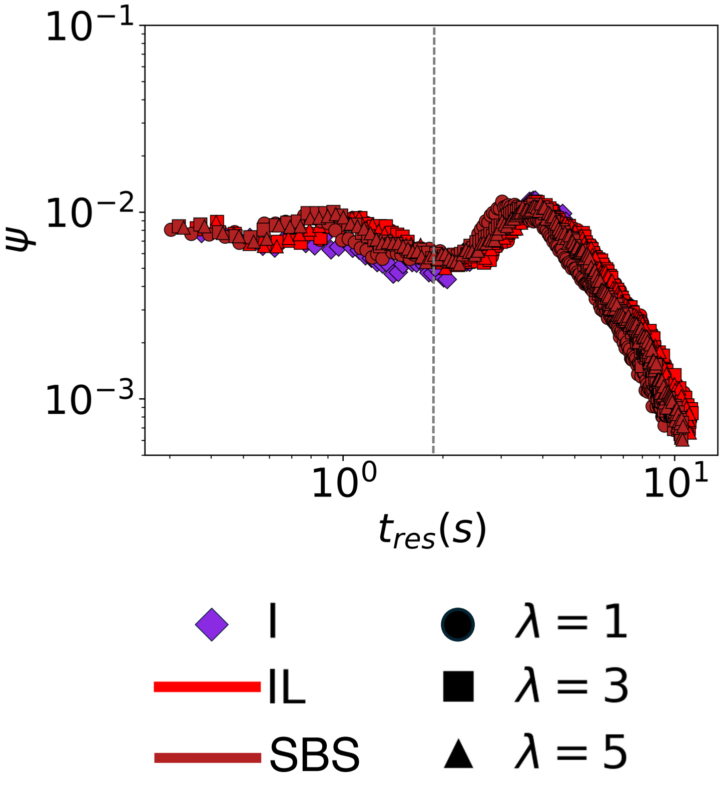

Figure S7 Distribution of the residence times for an isolated sponge (I), an upstream sponge in the IL configuration, and the right sponge with respect to the flow direction in the SBS configuration at $\mathrm{Re} = 500$. Results are reported for $\lambda = D, 3D$, and $5D$. The dashed gray line corresponds to the "ballistic" cross time $t_{\text{cross}} = \frac{D}{|\boldsymbol{u}_{in}|} \sim 1.9$ s at the considered flow regime.

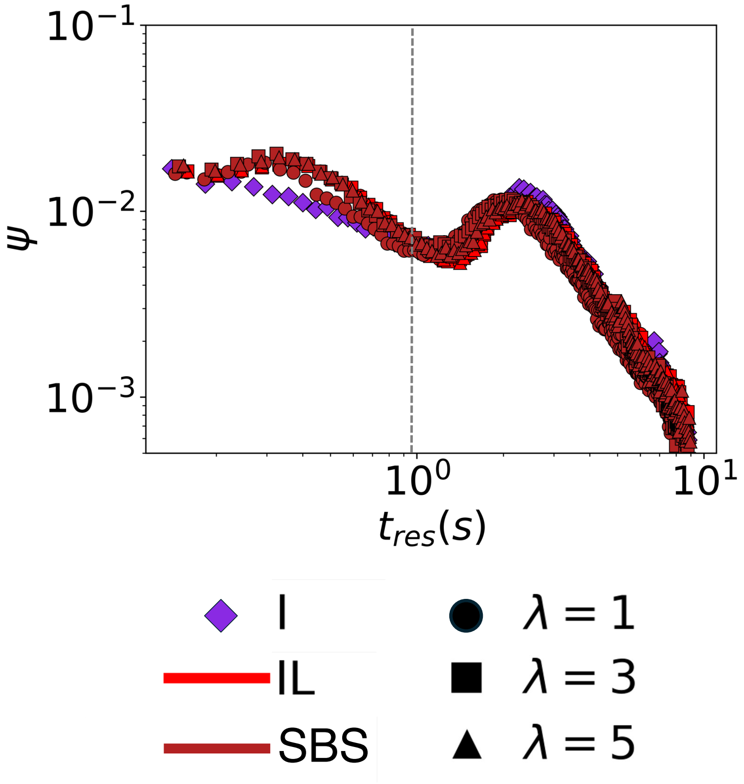

Figure S8 Distribution of the residence times for an isolated sponge (I), an upstream sponge in the IL configuration, and the right sponge with respect to the flow direction in the SBS configuration at $\mathrm{Re} = 1,000$. Results are reported for $\lambda = D, 3D$, and $5D$. The dashed gray line corresponds to the "ballistic" cross time $t_{\text{cross}} = \frac{D}{|\boldsymbol{u}_{in}|} \sim 0.95$ s at the considered flow regime.

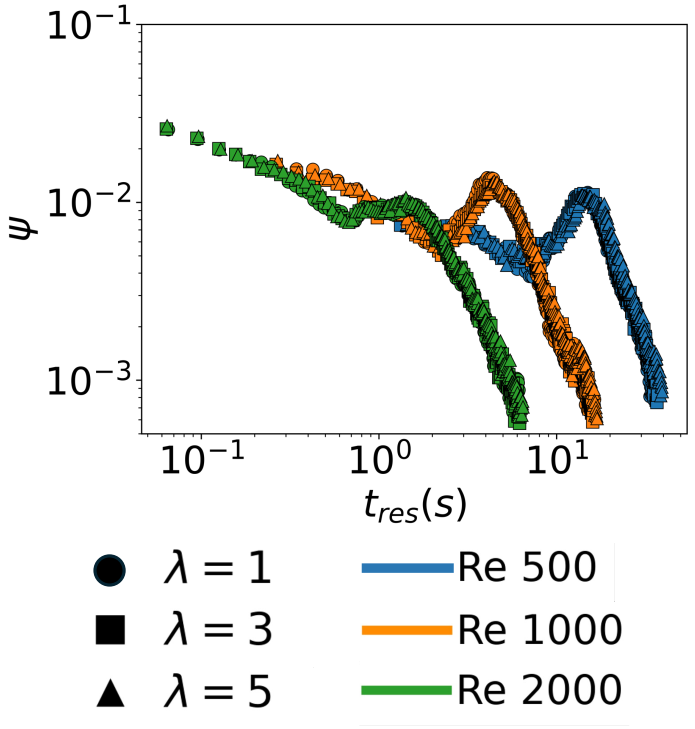

Figure S9 Distribution of the residence times in the left sponge, with respect to the flow direction, in the SBS configuration. Simulation results for $\mathrm{Re} = 500, 1,000$, and $2,000$, and $\lambda = D, 3D$, and $5D$.

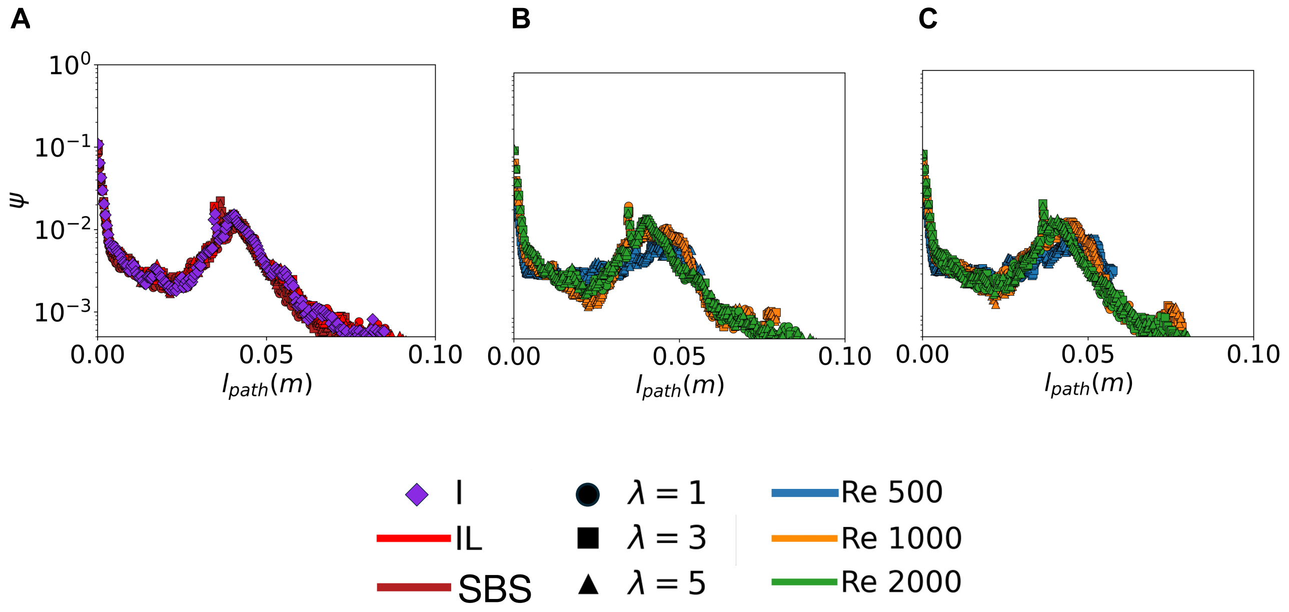

Figure S10 Distributions of pathline lengths. A) Comparison between the distribution of the pathline lengths between an isolated sponge (I), an upstream sponge in the IL configuration, and the right sponge with respect to the flow direction in the SBS configuration, at $\mathrm{Re} = 2,000$, for $\lambda = D, 3D$, and $5D$. B) Distribution of the pathline lengths in the upstream sponge in the IL configuration at $\mathrm{Re} = 500, 1,000$, and $2,000$ for $\lambda = D, 3D$, and $5D$. C) Distribution of the pathline lengths in the right sponge, with respect to the flow direction, in the SBS configuration at $\mathrm{Re} = 500, 1,000$, and $2,000$ for $\lambda = D, 3D$, and $5D$.

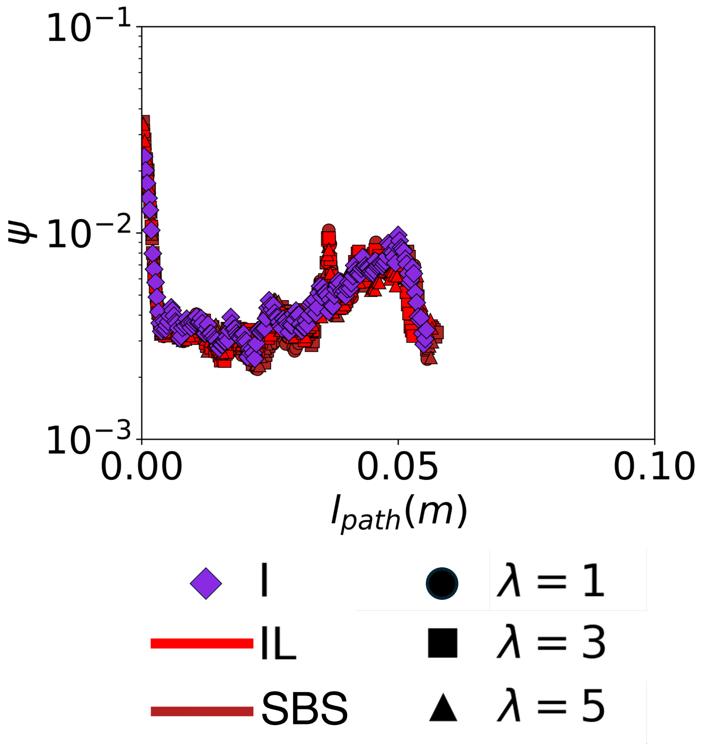

Figure S11 Distribution of the pathline lengths for an isolated sponge (I), an upstream sponge in the IL configuration, and the right sponge with respect to the flow direction in the SBS configuration at $\mathrm{Re} = 500$. Results are reported for $\lambda = D, 3D$, and $5D$.

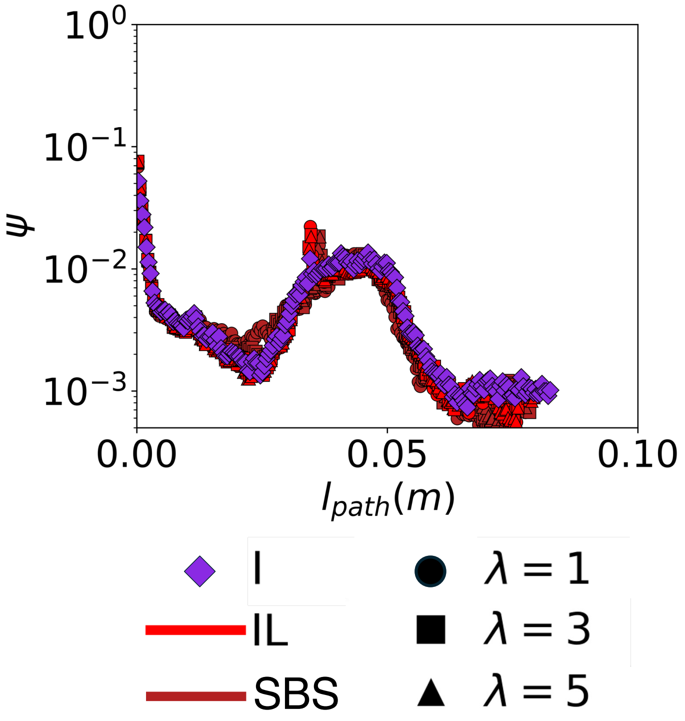

Figure S12 Distribution of the pathline lengths for an isolated sponge (I), an upstream sponge in the IL configuration, and the right sponge with respect to the flow direction in the SBS configuration at $\mathrm{Re} = 1,000$. Results are reported for $\lambda = D, 3D$, and $5D$.

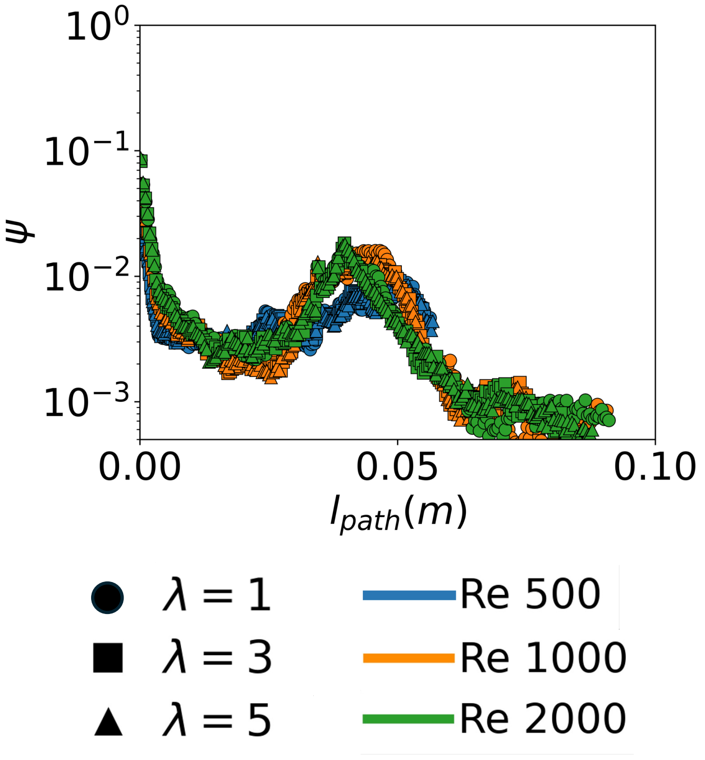

Figure S13 Distribution of the pathline length in the left sponge, with respect to the flow direction, in the SBS configuration. Simulation results for $\mathrm{Re} = 500, 1,000$, and $2,000$, and $\lambda = D, 3D$, and $5D$.

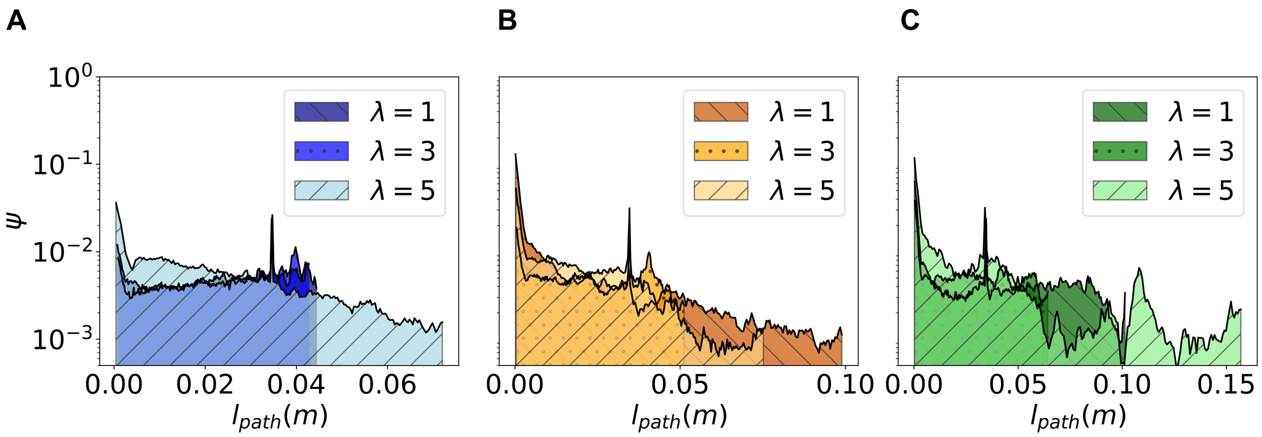

Figure S14 Distribution of pathline lengths within the body cavity of the downstream specimen of *E. aspergillum*. A) Distribution of the pathline lengths in the downstream sponge in the IL configuration at $\mathrm{Re} = 500$ for $\lambda = D, 3D$, and $5D$. B) Distribution of the pathline lengths in the downstream sponge in the IL configuration at $\mathrm{Re} = 1,000$ for $\lambda = D, 3D$, and $5D$. C) Distribution of the pathline lengths in the downstream sponge in the IL configuration at $\mathrm{Re} = 2,000$ for $\lambda = D, 3D$, and $5D$.

.

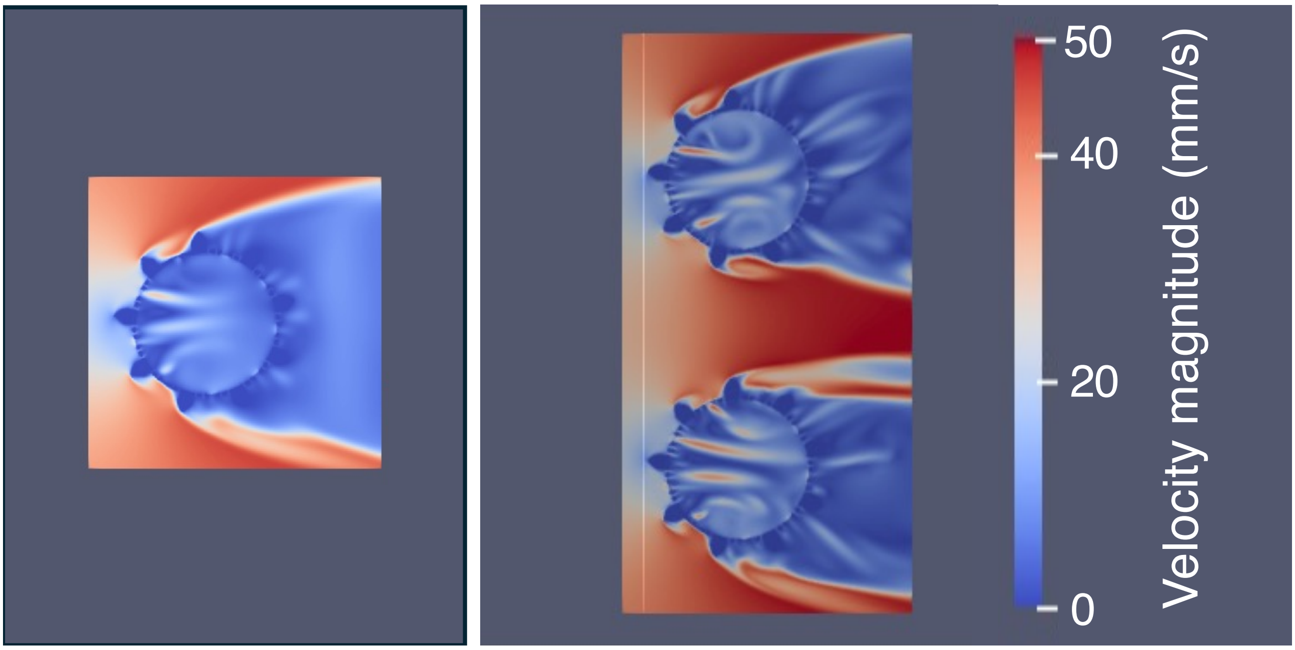

Figure S15 Velocity magnitude at $\mathrm{Re} = 1,000$ for isolated versus SBS sponges at $\lambda = D$. The same color legend is shown for both cases to emphasize increased water admission into the body cavity of each organism as compared to an isolated sponge.

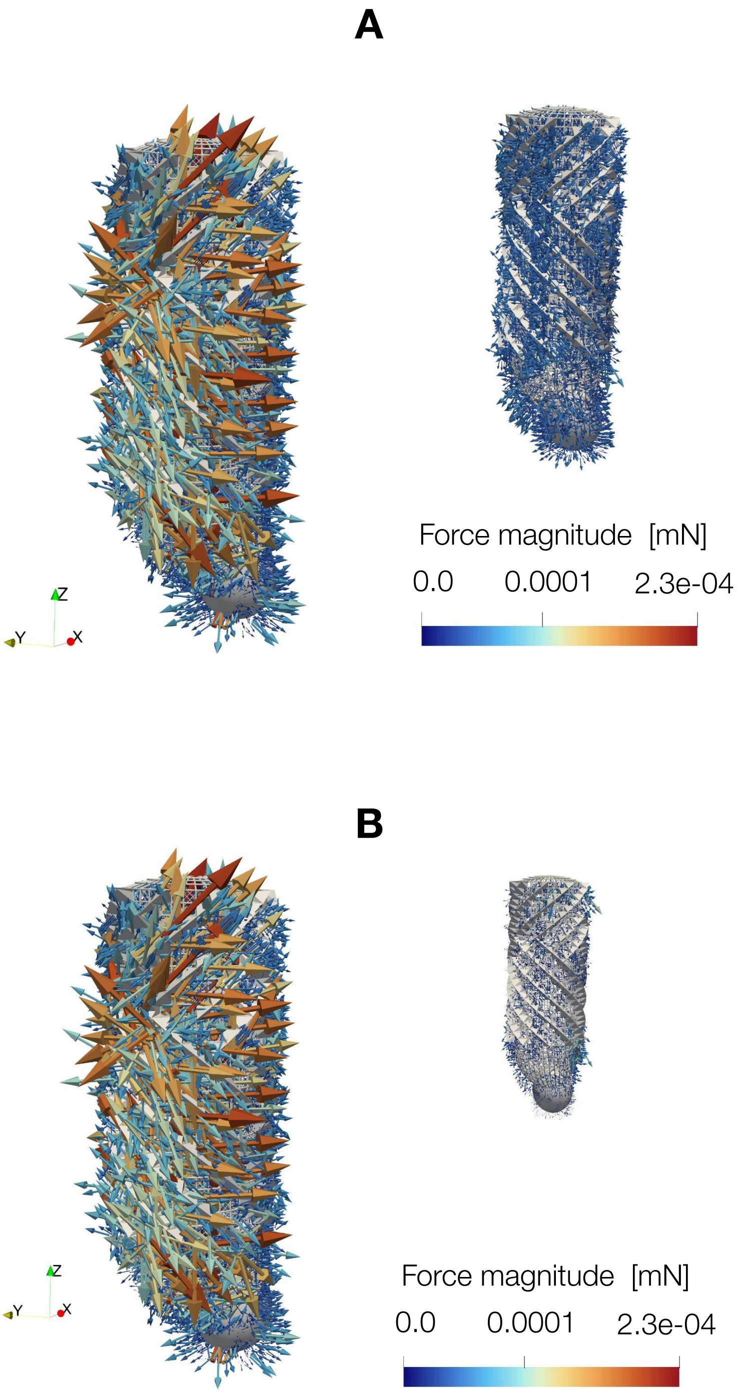

Figure S16 Forces acting on sponges IL at $Re = 2,000$ for different separation distances. A) Forces acting on the sponges at $\lambda = 3D$. B) Forces acting on the sponges at $\lambda = 5D$. Each vector is the force at a lattice grid point on the solid surface.

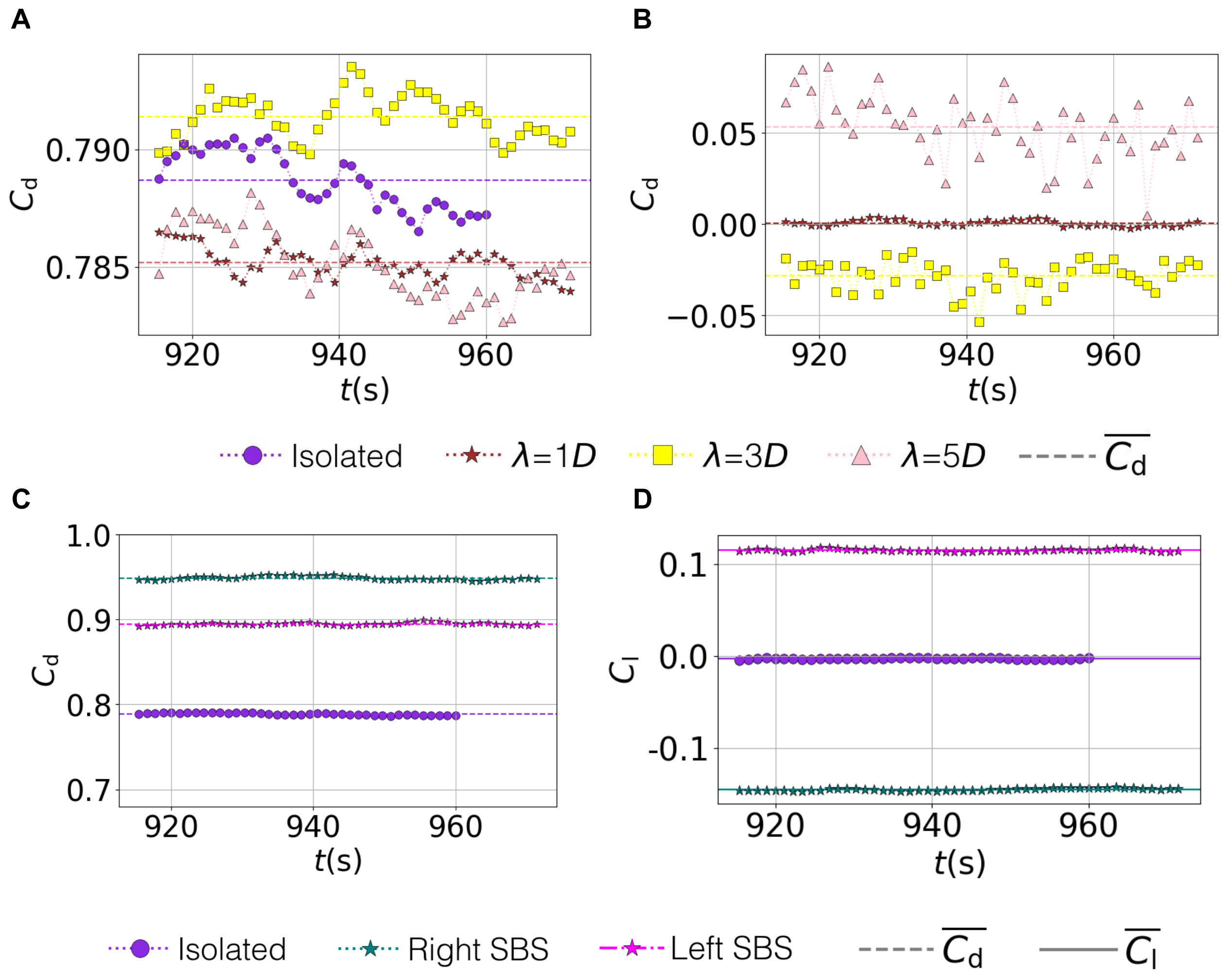

Figure S17 Time evolution of the drag and lift coefficient for different layouts and Reynolds numbers. A) Drag coefficient for an isolated sponge and the upstream sponge in the IL configuration for different separation distances at $\mathrm{Re} = 2,000$. B) Drag coefficient for the downstream sponge in the IL configuration for different separation distances at $\mathrm{Re} = 2,000$. C) Drag coefficient for an isolated sponge and left and right sponges in the SBS configuration for different separation distances at $\lambda = D$ and $\mathrm{Re} = 2,000$. D) Lift coefficient for an isolated sponge and left and right sponges in the SBS configuration for different separation distances at $\lambda = D$ and $\mathrm{Re} = 2,000$.

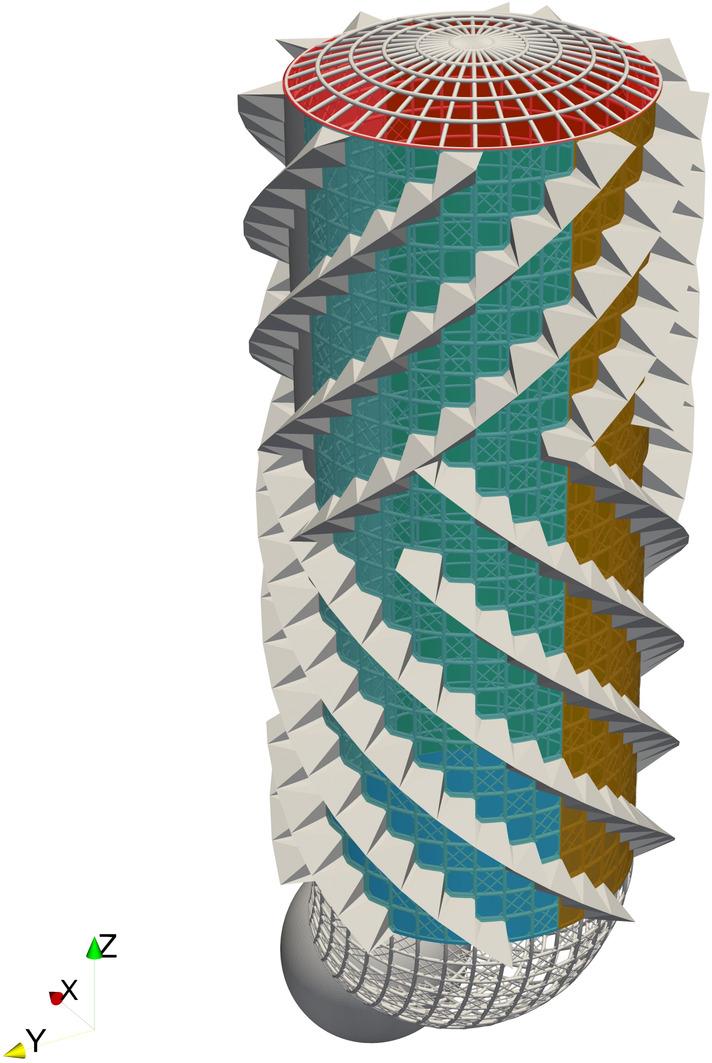

Figure S18 Sketch of the surfaces inside the body cavity used to evaluate water admission at different Re and λ.

Table S1 Comparison between the residence time in each of the IL sponges and the typical time for freely traversing a length equal to the sponge diameter. Percentage of grid points inside the body cavity characterized by a residence time, t_{res}, that is ten times larger than the time it would take for a particle to traverse a length equal to the sponge diameter, $t_{\text{cross}} = \frac{D}{|\boldsymbol{u}_{in}|}$.

Re	λ	Sponge 1 %	Sponge 2 %
500	1 D	39.92	100.00
	3 D	39.75	100.00
	5 D	39.29	99.17
1000	1 D	15.93	100.00
	3 D	16.08	100.00
	5 D	16.08	85.37
2000	1 D	7.31	100.00
	3 D	7.26	86.94
	5 D	7.70	52.11

Table S2 Drag force and drag coefficient for different layouts and $\mathrm{Re} = 500$. Configuration (first column), Reynolds number (second column), relative distance between the sponges scaled by their diameter (third column), drag force experienced by each sponge (fourth column), and drag coefficient (fifth column).

Configuration	Re	λ/D	$F_d(\mathrm{mN})$	C_d
Isolated sponge	500	0	1.07	0.60
Upstream IL	500	1	1.07	0.59
Upstream IL	500	3	1.07	0.60
Upstream IL	500	5	1.07	0.59
Downstream IL	500	1	-0.04	-0.02
Downstream IL	500	3	-0.10	-0.06
Downstream IL	500	5	-0.07	-0.04
Right SBS	500	1	1.33	0.74
Right SBS	500	3	1.21	0.68
Right SBS	500	5	1.17	0.65
Left SBS	500	1	1.20	0.67
Left SBS	500	3	1.19	0.66
Left SBS	500	5	1.11	0.62

Table S3 Drag force and drag coefficient for different layouts and $\mathrm{Re} = 1,000$. Configuration (first column), Reynolds number (second column), relative distance between the sponges scaled by their diameter (third column), drag force experienced by each sponge (fourth column), and drag coefficient (fifth column).

Configuration	Re	λ/D	$F_d(\mathrm{mN})$	C_d
Isolated sponge	1,000	0	4.95	0.69
Upstream IL	1,000	1	4.94	0.69
Upstream IL	1,000	3	4.93	0.69
Upstream IL	1,000	5	4.92	0.69
Downstream IL	1,000	1	-0.05	-0.01
Downstream IL	1,000	3	-0.18	-0.02
Downstream IL	1,000	5	-0.19	-0.03
Right SBS	1,000	1	5.98	0.83
Right SBS	1,000	3	5.39	0.75
Right SBS	1,000	5	5.17	0.72
Left SBS	1,000	1	5.60	0.78
Left SBS	1,000	3	5.38	0.75
Left SBS	1,000	5	5.19	0.72

Table S4 Drag force and drag coefficient for different layouts and $\mathrm{Re} = 2,000$. Configuration (first column), Reynolds number (second column), relative distance between the sponges scaled by their diameter (third column), drag force experienced by each sponge (fourth column), and drag coefficient (fifth column).

Configuration	Re	λ/D	$F_d(\mathrm{mN})$	C_d
Isolated sponge	2,000	0	22.61	0.79
Upstream IL	2,000	1	22.57	0.79
Upstream IL	2,000	3	22.66	0.79
Upstream IL	2,000	5	22.50	0.78
Downstream IL	2,000	1	-0.01	0.00
Downstream IL	2,000	3	-0.74	-0.03
Downstream IL	2,000	5	1.88	0.07
Right SBS	2,000	1	27.18	0.95
Right SBS	2,000	3	24.73	0.86
Right SBS	2,000	5	23.78	0.83
Left SBS	2,000	1	25.59	0.89
Left SBS	2,000	3	24.76	0.86
Left SBS	2,000	5	23.81	0.83

Table S5 Computational details for the simulations of an isolated sponge. Reynolds number (first column), Loop time (second column – defined as the time for one iteration), GLUPS (third column – representing the billion lattice updates per second), and GLUPS/GPU (fourth column – GLUPS per GPU).

Re	Loop time	GLUPS	GLUPS/GPU
500	26'137	3,232	4.90
1000	26'094	3,237	4.91
2000	32'099	2,631	3.99

Table S6 Computational details for the simulations of two IL sponges. Separation distance (first column), Reynolds number (second column), Loop time (third column – defined as the time for one iteration), GLUPS (fourth column – representing the billion lattice updates per second), and GLUPS/GPU (fifth column – GLUPS per GPU).

λ/D	Re	Loop time	GLUPS	GLUPS/GPU
1	500	26'214	3,222	4.88
1	1,000	26'159	3,229	4.89
1	2,000	26'189	3,224	4.88
3	500	26'223	3,220	4.87
3	1,000	26'121	3,234	4.89
3	2,000	26'223	3,221	4.88
5	500	26'079	3,239	4.91
5	1,000	26'071	3,240	4.91
5	2,000	26'164	3,229	4.89

Table S7 Computational details for the simulations of two SBS sponges. Separation distance (first column), Reynolds number (second column), Loop time (third column – defined as the time for one iteration), GLUPS (fourth column – representing the billion lattice updates per second), and GLUPS/GPU (fifth column – GLUPS per GPU).

λ/D	Re	Loop time	GLUPS	GLUPS/GPU
1	500	26'544	4,051	4.82
1	1,000	27'257	3,945	4.69
1	2,000	27'039	3,977	4.73
3	500	26'268	3,800	4.87
3	1,000	26'213	3,808	4.88
3	2,000	31'336	3,186	4.09
5	500	26'730	4,022	4.79
5	1,000	26'600	4,042	4.81
5	2,000	26'884	3,999	4.76
